# A photoacoustic finder fully integrated with a solid-state dye laser and transparent ultrasound transducer

**DOI:** 10.1016/j.pacs.2021.100290

**Published:** 2021-08-04

**Authors:** Byullee Park, Moongyu Han, Jeongwoo Park, Taejeong Kim, Hanyoung Ryu, Youngseok Seo, Won Jong Kim, Hyung Ham Kim, Chulhong Kim

**Affiliations:** a Department of Electrical Engineering, Convergence IT Engineering, Mechanical Engineering, and School of Interdisciplinary Bioscience and Bioengineering, Medical Device Innovation Center, Pohang University of Science and Technology, 77 Cheongam-Ro, Nam-Gu, Pohang, 37673, Republic of Korea; b Department of Chemistry, Postech-Catholic Biomedical Engineering Institute, School of Interdisciplinary Bioscience and Bioengineering, Pohang University of Science and Technology, 77 Cheongam-ro, Nam-gu, Pohang, 37673, Republic of Korea; c R&D center, Wontech Co. Ltd., Daejeon, 34028, Republic of Korea

**Keywords:** Photoacoustic sensing, Transparent ultrasound transducer, Solid-state dye laser, Melanoma, Sentinel lymph node

## Abstract

The standard-of-care for evaluating lymph node status in breast cancers and melanoma metastasis is sentinel lymph node (SLN) assessment performed with a handheld gamma probe and radioisotopes. However, this method inevitably exposes patients and physicians to radiation, and the special facilities required limit its accessibility. Here, we demonstrate a non-ionizing, cost-effective, handheld photoacoustic finder (PAF) fully integrated with a solid-state dye laser and transparent ultrasound transducer (TUT). The solid-state dye laser handpiece is coaxially aligned with the spherically focused TUT. The integrated finder readily detected photoacoustic signals from a tube filled with methylene blue (MB) beneath a 22 mm thick layer of chicken tissue. In live animals, we also photoacoustically detected both SLNs injected with MB and subcutaneously injected melanomas. We believe that our radiation-free and inexpensive PAF can play a vital role in SLN assessment.

## 1 Introduction

Because breast cancers and melanomas metastasize primarily through the lymphatic system [[1], [2], [3]], knowing the condition of the lymph nodes is essential in establishing the stage and prognosis of the cancer. It is also a key to effective local control of the disease [2]. A sentinel lymph node (SLN) is defined as the first gateway through which lymphatic drainage from the primary tumor passes. Thus, the SLNs are considered to reflect the status of the entire regional lymph branch. SLN biopsy, the standard-of-care for assessing lymph node conditions, is a safe surgery with low morbidity and low false-negative results [3]. It involves two procedures: localizing the SLN and dissecting it for pathological examination. SLN localization typically requires injecting a radioisotope (Technetium-99m) and a blue dye around the cancerous region [[2], [3], [4]]. The injected agents localize in the lymphatic system fairly quickly (*i.e.*, 30 min), and a handheld gamma probe is then used to sense the SLNs [5,6]. The blue dye helps to identify blue-colored lymph nodes with the naked eye post-incision. This combination of agents provides both high detection performance and a low false-negative rate [4]. However, the use of the radioisotope and gamma probe has three major drawbacks [2,7]: 1) Technetium-99m has a half-life of only 6 h (hr), so it must be injected in the nuclear medicine department, not in the operating room. 2) The radioisotope causes logistical problems, such as isotope handling and disposal. Its use also requires staff training and is subject to regulatory requirements. 3) Patients and physicians are potentially exposed to hazardous radiation.

To avoid these problems, near-infrared (NIR) fluorescence imaging of SLNs using non-radioactive indocyanine green (ICG) has been proposed [7]. Several studies have shown that the performance of ICG-based SLN localization is comparable to that of the radioisotope method [8,9]. However, this method suffers from a limited tissue penetration depth (1.0–1.5 cm) [10,11].

Photoacoustic (PA) imaging [[12], [13], [14], [15], [16], [17], [18], [19], [20], [21], [22], [23], [24], [25], [26], [27]]has also been explored as a non-ionizing and non-invasive method of identifying SLNs, using blue or green dyes [11,[28], [29], [30], [31]]. Advantageously, PA imaging can delineate SLNs in deep tissue with high resolution and high contrast. However, PA SLN imaging based on blue (methylene blue, MB) or green (indocyanine green, ICG) dyes requires expensive and bulky laser systems such as Q-switched Nd:Yag pumped optical parametric oscillators, Ti: Sapphire lasers, or liquid dye lasers [[32], [33], [34], [35], [36], [37], [38], [39], [40], [41]]. Thus, these systems are not yet widespread in clinical settings.

Here, we propose a handheld, non-ionizing, and cost-effective PA finder (PAF) to localize SLNs. The PAF fully integrates a custom transparent ultrasound transducer (TUT) and a solid-state dye laser handpiece. Unlike a conventional opaque UT, the TUT seamlessly enables coaxial optical and acoustic beams in a small form factor [42]. Our TUT is the first to use a single crystal lead magnesium niobate-lead titanate (PMN-PT), different from the TUT previously developed, which uses lithium niobate (LNO) [42] and polyvinylidene fluoride (PVDF) [43]. As a piezoelectric material, PMN-PT is especially suitable in the low-frequency range, due to its high electromechanical and dielectric properties compared to LNO and PVDF [44]. In *in vitro* experiments, we detected PA signals from a tube filled with MB beneath a 22 mm thickness of chicken tissue. In *in vivo* experiments, after intradermal injection of MB, we sensed PA signals from SLNs in rats. Subsequently, we acquired PA signals from subcutaneously injected melanoma in living mice. In all the *in vivo* experiments, the PA signals were detected from beneath an 18 mm thickness of chicken tissue.

## 2 Materials and methods

### 2.1 Fabrication of transparent ultrasound transducer

**Fig. 1:**
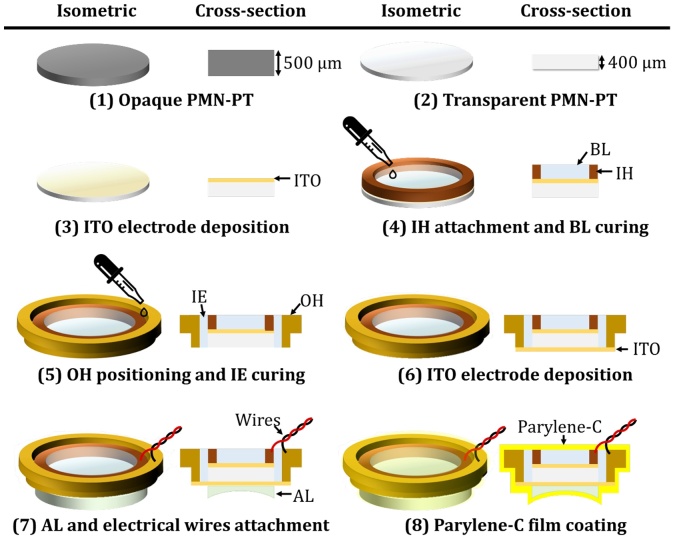
Fabrication of a transparent ultrasound transducer (TUT). PMN-PT, lead magnesium niobate-lead titanate; ITO, indium tin oxide; BL, backing layer; IH, inner housing; IE, insulation epoxy; OH, outer housing; AL, acoustic lens.

Fig. 1a shows the eight fabrication steps of the TUT:

1) A 6 mm diameter circular disk of 500 μm thick conventional opaque <001 > DC poled PMN-PT single crystal [45,46] (CTS Corp., USA) was prepared. This material was selected for its high electromechanical coupling coefficient ($k_{t}$ ≅ 0.60), high longitudinal velocity (c ≅ 4600 m/s), high piezoelectric constant ($d_{33}$ ≅ 1500 pC/N), and high transparency (80 % at 650 nm).
2) The PMN-PT was transparently lapped to a thickness of 400 μm using a precision lapping and polishing system (PM6, Logitech LTD, UK). Both sides of the opaque PMN-PT were gently polished using fine calcined aluminum oxide powder with powder sizes from 3 μm to 30 μm. No additional DC poling was performed because the entire process of making the PMN-PT transparent was kept below 65 °C, much lower than the 130 °C temperature at which the piezoelectric properties of PMN-PT are thermally depoled [47,48].
3) As a transparent electrode, a 200 nm thickness of indium-tin-oxide (ITO) was deposited on the upper surface of the PMN-PT.
4) A ring-shaped inner housing (IH), acting as an electrical bridge, was bonded to the ITO layer using conductive epoxy (E-solder 3022, Von Roll, Switzerland). Then, to form a backing layer (BL), degassed Epo-Tek-301epoxy (Epoxy Technology, Inc., USA) was filled inside the IH and cured at room temperature for 24 h. The BL damps unnecessary acoustic signals.
5) An outer housing (OH) was placed concentrically on the IH, and the gap between the OH and IH was filled with insulating epoxy (IE, S-209, Devcon Ltd., USA).
6) To form an electrical ground, ITO was deposited on the bottom of the PMN-PT and OH.
7) To focus the acoustic field, an acoustic lens (AL, #48−267, Edmund Optics, USA) was attached under the ITO layer, using an ultraviolet light-cured adhesive (Norland Optical Adhesive 81, Norland Products, Inc., USA). Further, electrical wires were connected to the IH and OH using the conductive epoxy.
8) A parylene-C film with a thickness of 5 μm was deposited on the entire outer surface of the TUT, using a parylene deposition system (PDS 2010, Specialty Coating Systems, Inc., USA).

### 2.2 Optical and acoustical properties of the transparent ultrasound transducer

**Fig. 2:**
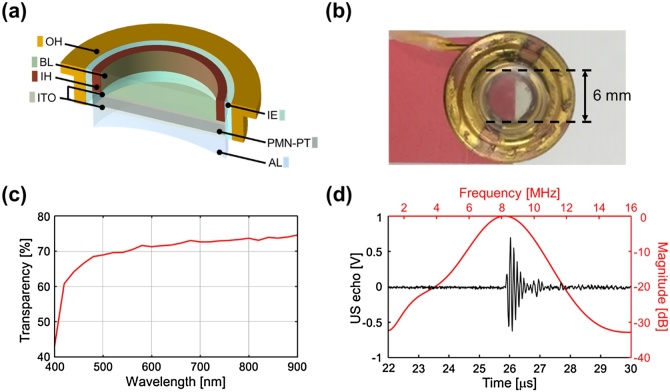
Characteristics of a transparent ultrasound transducer (TUT). (a) Layer-by-layer view and (b) a photograph of the TUT. (c) Optical transparency of the TUT. (d) Pulse-echo response and spectrum of the TUT. OH, outer housing; BL, backing layer; IH, inner housing; ITO, indium tin oxide; IE, insulation epoxy; PMN-PT, lead magnesium niobate-lead titanate; AL, acoustic lens; and US, ultrasound.

Fig. 2a shows each layer of the TUT, and Fig. 2b, a photograph of the TUT, shows its active diameter of 6 mm above red and white paper to demonstrate its transparency. The optical transparency of the TUT was measured at wavelengths from 400 to 900 nm (Fig. 2c). The TUT shows transparency of 72 % at 650 nm. To evaluate the performance of the TUT, a pulse-echo response was measured by using a pulser–receiver (P/R) (DPR300, JSR Corp., Japan) (Fig. 2d). The measured center frequency was 8.0 MHz, and the -6 dB fractional bandwidth was 45 %. This frequency band of the TUT, 6–10 MHz, is mainly used for pre-clinical and clinical SLN imaging or sensing [31,[49], [50], [51], [52], [53]]. The AL extended the acoustic focal length of the TUT to 19 mm. In addition, the electrical impedance of the TUT was measured using an impedance analyzer (E4990A, Keysight, USA) and k_t_ is calculated to be 0.57 (Supplementary Fig. S2).

### 2.3 Handheld photoacoustic finder

**Fig. 3:**
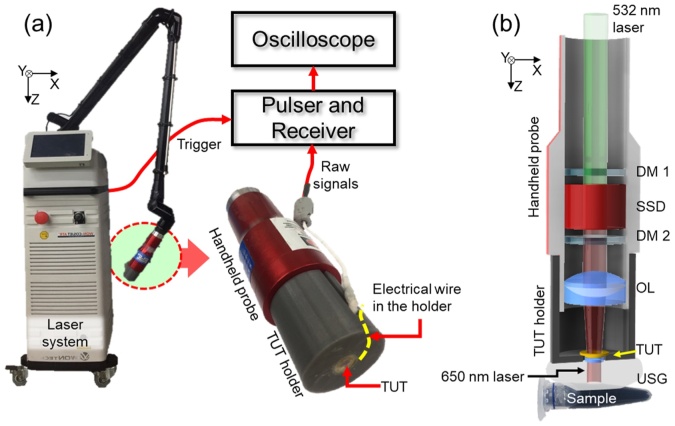
Schematic of the handheld photoacoustic finder system. (a) The portable solid-state dye laser system with a handheld probe. (b) Detailed schematic of the handheld probe. TUT, transparent ultrasound transducer; DM, dichroic mirror; SSD, solid-state dye; OL, objective lens; and USG, ultrasound gel.

The handheld PAF was developed by integrating the TUT with a commercial portable laser system (COSJET ATR, WONTECH Corp., Republic of Korea). As Fig. 3 shows, the laser system consists of a portable Nd:YAG laser body (288 mm × 583 mm × 847 mm in X—YZ—) and an articulated arm with a solid-state dye handpiece (HOYA ConBio 650 Dye Handpiece, Laser Service Solutions, USA) (Fig. 3a). The laser system generates 532 nm pulsed light with a pulse duration of 5–40 ns and a repetition rate of up to 10 Hz. Then, the laser pulse is transmitted through the articulated arm to the dye handpiece. The TUT holder integrates the handpiece and the TUT coaxially. The PA signals sensed *via* the TUT are amplified through a P/R and displayed on an oscilloscope. The PAF handpiece measures 38.5 mm × 38.5 mm × 140 mm (X—YZ—), small enough for handheld operation (Fig. 3b). A solid-state dye between two dichroic mirrors changes the light wavelength from 532 nm to 650 nm, a desirable wavelength for sensing MB and melanoma. The light output is delivered to the sample through a confocally placed objective lens with a focal length of 30 mm and the TUT. An ultrasound (US) gel placed between the TUT and the sample provides acoustic coupling.

### 2.4 *In vitro* and *in vivo* experiments

All animal experiments were conducted according to the Pohang University of Science and Technology guidelines for the care and use of laboratory animals. The laser fluence for all *in vivo* experiments was set to 15 mJ/cm^2^ which is below the safe exposure limits for skin as standardized by the American National Standards Institute (ANSI) (*i.e.*, 20 mJ/cm^2^) [54]. For the *in vivo* SLN localization experiments, a vaporized-isoflurane system (delivering 1 L/min of oxygen and 0.75 % isoflurane) was used to initially anesthetize a healthy rat (Sprague Dawley, female, 4 weeks, ∼130 g) and maintain anesthesia during the experiments. To localize the SLN, 0.1 mL of MB with a concentration of 30 mM was used. As a second *in vivo* demonstration, cutaneous melanoma detection experiments on mice were conducted. For *in vivo* melanoma tumor model preparation, B16-F1 melanoma cells (Korean Cell Line Bank, 5 × 10^4^ cell/mouse) were subcutaneously injected into the right flank of female BALB/c-nu/nu mice (8 weeks, ∼22 g). The cells were cultured in Dulbecco’s modified Eagle’s medium (DMEM) with 10 % fetal bovine serum (FBS), 100 U ∙ mL^−1^ penicillin, and 100 μg ∙ mL^−1^ streptomycin, incubated at 37 °C in a 5% CO_2_ humidified incubator.

## 3 Results

### 3.1 *In vitro* deep-tissue photoacoustic signal detection

**Fig. 4:**
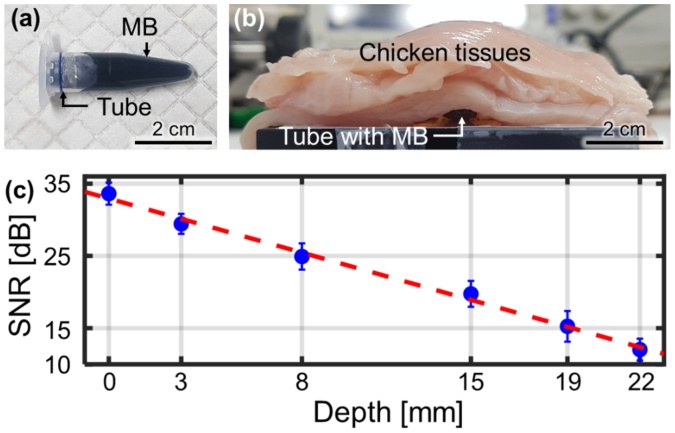
*In vitro* deep tissue PA signal detection experiment. (a) A sample tube filled with MB. (b) The tube placed under 4 slices of chicken tissue. (c) PA SNR *vs* depth. PA, photoacoustic; SNR, signal-to-noise ratio; and MB, methylene blue. (For interpretation of the references to colour in the Figure, the reader is referred to the web version of this article).

To explore the PA signal sensing capability of the PAF, we conducted *in vitro* deep tissue PA sensing experiments (Fig. 4). As a target, a 1-mL microtube was filled with MB of 30 mM (Fig. 4a). As seen in Fig. 4b, five slices of chicken tissue, making a total thickness of 22 mm, were successively laid atop the microtube. The 650-nm laser fluence for this *in vitro* experiment was 14 mJ/cm^2^, below the ANSI laser safety limit of 20 mJ/cm^2^ [54]. The PA signal was first measured without chicken tissue, and the signal-to-noise (SNR) was 33 dB (Fig. 4c). The error bar denotes the standard deviation (n = 3). Subsequently, PA signals were measured while incrementally stacking the chicken tissue layers. As the sensing depth increases, the SNR decreases exponentially. The maximum depth at which PA signals could be detected is 22 mm, where the SNR of 12 dB. The effective attenuation coefficient (μ_eff_) of the system, 1.1 cm^−1^, was calculated from the slope of the fitted line of SNR *versus* depth, and the calculated effective penetration depth was 0.92 cm. The noise equivalent depth is 34 mm, which is 3.7 times deeper than the 1/e penetration depth of 0.92 cm.

### 3.2 *In vivo* photoacoustic sentinel lymph node localization

**Fig. 5:**
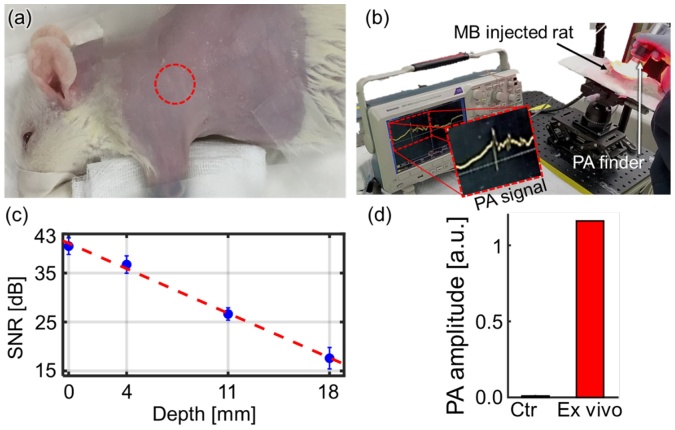
*In vivo* PA SLN localization experiments using intradermally injected methylene blue in rats (n = 3). (a) Photograph of the rat. (b) Snapshot from the SLN localization experiment video (Supplementary Video S1). (c) PA SNRs estimated at different depths of 0, 4, 11 and 18 mm. (d) *Ex vivo* PA amplitudes of the dissected SLNs. SLN, sentinel lymph node; PA, photoacoustic; and SNR, signal-to-noise ratio. (For interpretation of the references to colour in the Figure, the reader is referred to the web version of this article).

After the hair was removed from the left axillary area of the rat (Fig. 5a), the MB, described above, was intradermally injected into the left forefoot using a 30-gauge syringe. The red dashed circle indicates the PA sensing region where the SLN is presumed to be. We started detecting PA signals in the SLN 10 min after MB injection. The experimental process was recorded as a video (Supplementary Video S1), and Fig. 5b shows a snapshot from the video. A strong time-resolved PA signal with a magnitude 1.1 V_peak-to-peak_ is observed at the MB-localized SLN. We initially photoacoustically localized the SLN without overlying chicken tissue and calculated the PA SNR to be 40 dB (Fig. 5c). The error bar denotes the standard deviation (n = 3). To confirm the ability to localize deep tissue SLNs *in vivo*, three chicken tissue slices were stacked layer by layer above the SLN, and PA signals were detected from beneath each layer. The SNR exponentially decreases as the imaging depth increases. The maximum PA sensing depth is 18 mm, at which the SNR of 18 dB. In *ex vivo* validation experiments, the MB-containing lymph node and a normal one from the other side were dissected and photoacoustically compared (Fig. 5d). The drainage SLN provided greatly increased PA amplitude. These results support the usefulness of the handheld PAF as a non-invasive, non-ionizing tool for lymph node localization.

### 3.3 *In vivo* photoacoustic cutaneous melanoma detection

**Fig. 6:**
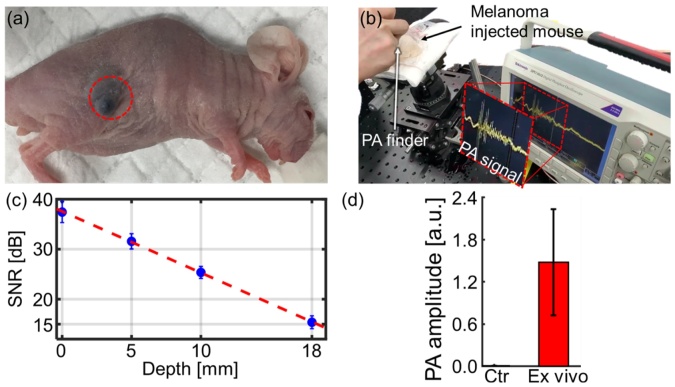
*In vivo* PA detection of subcutaneously injected melanoma in mice (n = 3). (a) Photograph of the mouse, with melanoma circled in red. (b) Snapshot from video recording of the experiments (Supplementary Video S2). (c) PA SNRs estimated from beneath sequentially overlaid tissue depths of 0, 5, and 10 mm. (d) *Ex vivo* PA amplitude of the dissected melanoma tissue. PA, photoacoustic, and SNR, signal-to-noise ratio. (For interpretation of the references to colour in the Figure, the reader is referred to the web version of this article).

To further explore possible applications of PAF, we performed *in vivo* cutaneous melanoma detection experiments on mice. Because melanoma itself highly absorbs light in the visible and NIR ranges, a strong PA signal can be obtained without dye injection. One week after the melanoma inoculation, the tumor volume had reached 150 mm^3^, and the mouse was anesthetized using the vaporized-isoflurane system (Fig. 6a). The melanoma detection process was video recorded (Supplementary Video S2), and Fig. 6b is a snapshot from the video. The PAF detected a strong time-resolved PA signal with a magnitude of 1 V_peak-to-peak_ from the melanoma. We photoacoustically detected PA signals from the melanoma site indicated by the red circle and confirmed the SNR of 37 dB (Fig. 6c). To demonstrate the capability to sense deep tissue melanoma *in vivo*, three chicken tissue layers were successively stacked and PA signals were detected from beneath each thickness. The maximum penetration depth is 18 mm with a PA SNR of 15 dB. The error bar denotes a standard deviation (n = 3). After sacrificing the mice, the PA signals were detected from the dissected melanoma-containing tissue and normal tissue of the opposite flank. From the dissected melanoma tissue, we obtained a much stronger PA signal than normal tissue (Fig. 6d). These results show that our system can sense melanoma-specific targets and thus can be further applied to melanoma SLN biopsy.

## 4 Discussion and conclusion

Although ICG-based fluorescence localization of SLN has emerged as an alternative that overcomes problems caused by radioisotopes, but its clinical use is hampered by the insufficient penetration depth of the NIR camera. High-resolution PA sensing, which is similarly non-radioactive and non-invasive, avoids this shortcoming, and PA sensing or imaging studies for SLN biopsy have been successfully conducted on small animals and humans [11,29,51]. However, conventional PA imaging of SLNs requires a multi-channel system that can be expensive and bulky. Moreover, physicians familiar with gamma probes need additional time and effort to adapt to a new clinical imaging protocol.

To our best knowledge, our proposed photoacoustic finder (PAF) system is the first handheld PA sensing tool for SLN localization. Similar in size and use to a gamma probe, it is relatively easy to translate to clinics. Laser safety concerns are minimal because the solid-state dye laser system has been routinely used in dermatology. The system’s key component, the TUT, overcomes the structural limitations of conventional PA imaging or sensing systems. Thanks to the transparency of the transducer, the optical path and TUT are confocally aligned. In addition, the AL effectively focuses the acoustic field of the TUT. These features minimize the system’s size and improve its SNR. Here, we detected PA signals *in vitro* from an MB-filled tube beneath a 22 mm thickness of chicken tissue. In *in vivo* experiments, after intradermal injection of MB, we photoacoustically localized SLNs in rats, and we also sensed PA signals from subcutaneously injected melanoma in mice without exogenous agents. In addition, the concentration of MB-localized SLN of a rat injected with 0.1 mL of 30 mM MB is approximately 7 mM [55]. In contrast, the average hemoglobin concentration in the blood vessels is 2.32 mM [56]. As a result of calculating absorption coefficients based on these concentrations, the MB, deoxy-hemoglobin (Hb) and oxy-hemoglobin (HbO2) were 1010 cm^−1^, 20 cm^−1^, and 47.1 cm^−1^, respectively (Supplementary Fig. S2). To demonstrate that MB-localized SLN can be distinguished from hemoglobin using PAF, we combined PAF with a 2-axis motorized scanner to obtain a two-dimensional SLN PA image from a MB-injected rat. As a result, the MB-localized SLN in the rat was dominantly differentiated through the two-dimensional PA image (Supplementary Figs. S3). Further, the PA amplitude in the plastic tube containing MB was 9-folds stronger than that in the plastic tube containing fresh rat blood (Supplementary Figs. S4). We also sensed PA signals from subcutaneously injected melanoma in mice without exogenous agents. In all *in vivo* experiments, the PA signals were detected after stacking 18-mm thick chicken tissue. Potentially, our handheld PAF can evaluate cancer metastasis by performing SLN assessment without a gamma probe, solving various problems caused by the use of radioactive isotopes. Furthermore, we expect that SLN assessments of cancers such as melanoma that have their own pigment can be performed without dye injection.

In human beings, SLNs are mainly located 1.2 ± 0.5 cm from skin [55]. According to the previous literatures, the 1/e optical penetration depths in human breast tissue and chicken breast tissue are 0.78 cm [55]and 1.1 cm [50], respectively. This is comparable to the calculated 1/e penetration depth of 0.92 cm based on our experimental results. In previous studies with similar 1/e penetration depths to ours [51,55], PA SLN images were obtained from human breast tissue and chicken breast tissue from sufficient depth. Although we showed the feasibility of the handheld PAF for SLN localization at a depth of 18 mm, this sensing depth is not sufficient for human studies, since PA signals in human tissues are more attenuated than in chicken breast tissues (0.78 cm *vs*. 1.1 cm) due to the optical absorption factors such as blood and lipids. Therefore, assessing the sensing depth using a living tissue will yield more accurate results. To compensate this, following approaches can be pursued in the future: 1) Develop a lower frequency of the TUT (4–5 MHz). The attenuation coefficient is inversely proportional to frequency and distance. The attenuation of soft tissue and fat is 0.75 dB/cm/MHz [57]. Replacing the 8 MHz TUT with a 4 MHz TUT prevents a 5.4 dB of acoustic attenuation loss in soft tissue 18 mm deep. 2) Double the aperture size of the TUT. The sensitivity of a piezoelectric detector is proportional to its element size [58]. Increasing the current TUT aperture diameter from 9 mm to 18 mm could give a 4-folds improvement in sensitivity. 3) Average the PA signal. Averaging is one of the simplest ways to efficiently reduce random noise while maintaining the PA response [59]. Although averaging was not applied in our study due to the slow PRF of the laser source (up to 10 Hz), averaging can be performed using a relatively high-speed laser to effectively increase the SNR in the subsequent study. 4) Use a proper matching layer to improve the acoustic energy transfer efficiency. The acoustic energy transfer efficiency can be improved by replacing the acoustic lens with an acoustic impedance of 11–13 MRayl we used with a material with an acoustic impedance of 7–8 MRayl [44]. 5) Raise the laser fluence to near the ANSI limit (20 mJ/cm^2^) to maximize the light penetration depth in the tissue. In the future, such system improvements are expected to enable SLN localization and melanoma detection in biological tissues 3−5 cm deep *in vivo*.

## Declaration of Competing Interest

Chulhong Kim has financial interests in OPTICHO, which, however, did not support this work.
