# Supplementary material for: A photoacoustic finder fully integrated with a solid-state dye laser and transparent ultrasound transducer
Source: Photoacoustics. 2021 Aug 4;23:100290. doi: 10.1016/j.pacs.2021.100290 (PMC8358697; doi:10.1016/j.pacs.2021.100290)

**Supplementary information for:**

**Manuscript number: PACS_D_21-00048**

**Manuscript title: A Photoacoustic Finder Fully Integrated with a Solid-State Dye Laser and Transparent Ultrasound Transducer**

Byullee Park^a,†^, Moongyu Han^a,†^, Jeongwoo Park^a,†^, Taejeong Kim^b^, Hanyoung Ryu^c^, Youngseok Seo^c^, Won Jong Kim^b^, Hyung Ham Kim^a,^*, and Chulhong Kim^a,^*

^a^Department of Electrical Engineering, Convergence IT Engineering, Mechanical Engineering, and School of Interdisciplinary Bioscience and Bioengineering, Medical Device Innovation Center, Pohang University of Science and Technology, 77 Cheongam-Ro, Nam-Gu, Pohang 37673, Republic of Korea

^b^Department of Chemistry, Postech-Catholic Biomedical Engineering Institute, School of Interdisciplinary Bioscience and Bioengineering, Pohang University of Science and Technology, 77 Cheongam-ro, Nam-gu, Pohang, 37673, Republic of Korea

^c^R&D center, Wontech Co. Ltd., Daejeon 34028, Republic of Korea

^†^These authors contributed equally to this work.

*Corresponding authors: [chulhong@postech.edu](mailto:chulhong@postech.edu) and [david.kim@postech.ac.kr](mailto:david.kim@postech.ac.kr)

**Table of contents**

**Supplementary Figures**

Fig. S1. Measured electrical impedance and phase of the TUT1

Fig. S2. Optical absorption coefficient of MB, Hb, and HbO_2_2

Fig. S3. *In vivo* two-dimensional PA imaging from a methylene blue injected rat.3

Fig. S4. Comparison of PA amplitude in MB vs. rat blood *in vitro*4

.


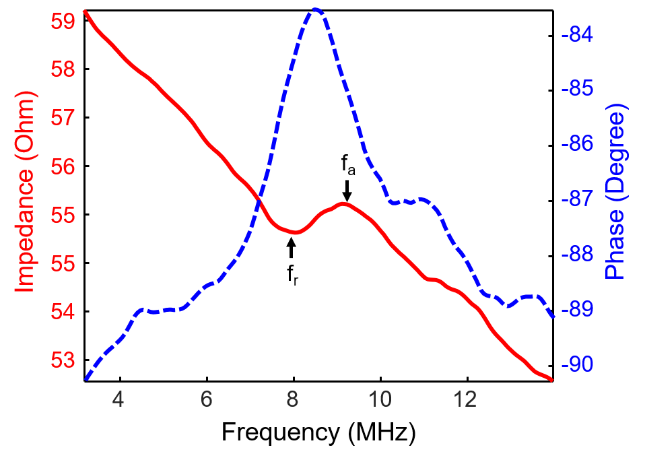


**Supplementary Fig. S1.** Measured electrical impedance and phase of the TUT.　f_r_ indicates resonant frequency peak, and f_a_ indicates anti-resonant frequency peak. TUT, transparent ultrasound transducer

**Supplementary Fig. S2.** Optical absorption coefficient of MB with a concentration of 7 mM, Hb and HbO_2_ with a concentration of 2.32 mM. MB, methylene blue; Hb, deoxy-hemoglobin; HbO2, oxy-hemoglobin.

**Supplementary Fig. S3.** *In vivo* two-dimensional PA imaging from a methylene blue injected rat. (a) Photograph of a rat intradermally injected with MB into the left forefoot. A red dashed box (5 mm x 5 mm) indicates the PA scan region where the SLN is expected to be localized. (b) PA image acquired in the red dashed box in panel a. (c) Photograph of MB-localized SLN with tissue removed after scanning. PA, photoacoustic; SLN, sentinel lymph node.


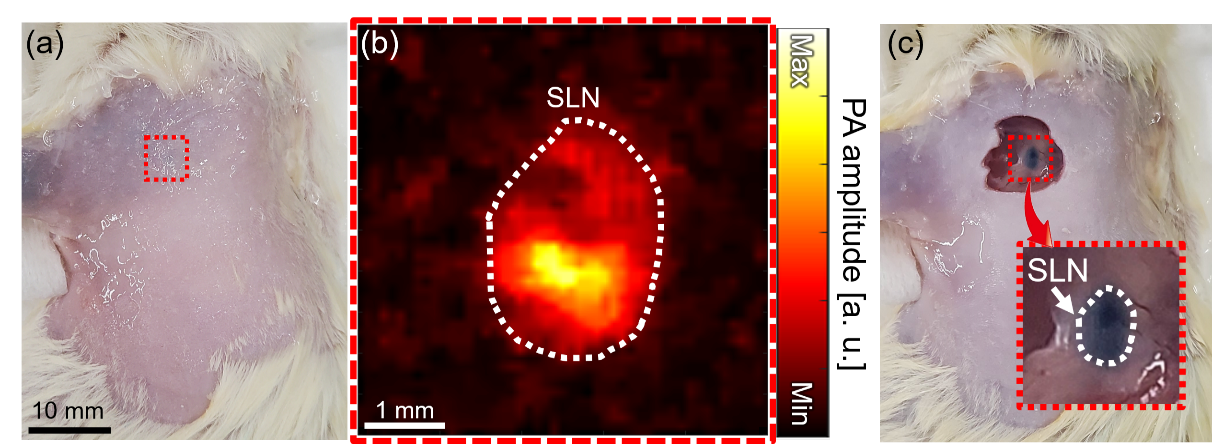


**Supplementary Fig. S4.** Comparison of PA amplitude in MB vs. rat blood *in vitro*. (a) Tubes filled with 30 mM MB and fresh rat blood. (b) Quantification of the PA amplitude obtained from the MB and rat blood sample of panel a using the PAF. PA, photoacoustic; MB, methylene blue and PAF, photoacoustic finder.


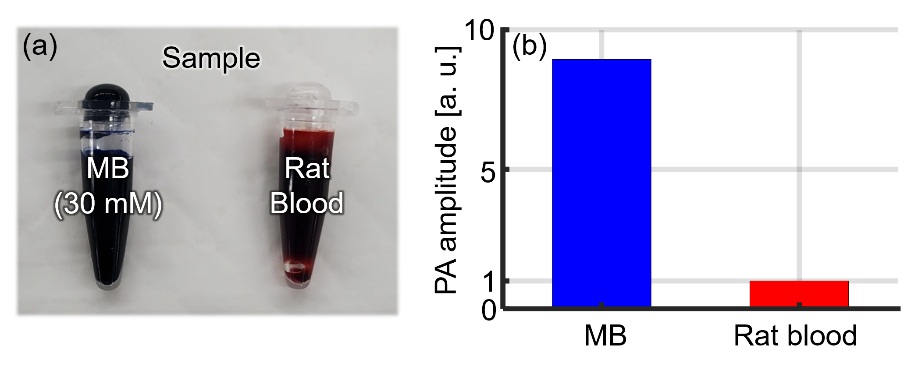

Supplement: Supplementary file 1 [file mmc1.docx]
